# Synthesis of CuO and PAA-Regulated Silver-Carried CuO Nanosheet Composites and Their Antibacterial Properties

**DOI:** 10.3390/polym14245422

**Published:** 2022-12-11

**Authors:** Zhihui Ni, Menghui Wan, Gongming Tang, Lei Sun

**Affiliations:** 1 Center for Advanced Materials Research, Zhongyuan University of Technology, Zhengzhou 450007, China; 2 Engineering Research Center for Nanomaterials, Henan University, Kaifeng 475004, China

**Keywords:** CuO@Ag, nanosheet composite, facile and green approach, antimicrobial property, synergy

## Abstract

With the aid of a facile and green aqueous solution approach, a variety of copper oxide (CuO) with different shapes and polyacrylic-acid (PAA)-regulated silver-carried CuO (CuO@Ag) nanosheet composites have been successfully produced. The point of this article was to propose a common synergy using Ag-carried CuO nanosheet composites for their potential antibacterial efficiency against three types of bacteria such as *E. coli*, *P. aeruginosa*, and *S. aureus*. By using various technical means such as XRD, SEM, and TEM, the morphology and composition of CuO and CuO@Ag were characterized. It was shown that both CuO and CuO@Ag have a laminar structure and exhibit good crystallization, and that the copper source and reaction duration have a sizable impact on the morphology and size distribution of the product. In the process of synthesizing CuO@Ag, the appropriate amount of polyacrylic acid (PAA) can inhibit the agglomeration of Ag NPs and regulate the size of Ag at about ten nanometers. In addition, broth dilution, optical density (OD 600), and electron microscopy analysis were used to assess the antimicrobial activity of CuO@Ag against the above three types of bacteria. CuO@Ag exhibits excellent synergistic and antibacterial action, particularly against *S. aureus*. The antimicrobial mechanism of the CuO@Ag nanosheet composites can be attributed to the destruction of the bacterial cell membrane and the consequent leakage of the cytoplasm by the release of Ag^+^ and Cu^2+^. The breakdown of the bacterial cell membrane and subsequent leakage of cytoplasm caused by Ag^+^ and Cu^2+^ released from antimicrobial agents may be the cause of the CuO@Ag nanosheet composites’ antibacterial action. This study shows that CuO@Ag nanosheet composites have good antibacterial properties, which also provides the basis and ideas for the application research of other silver nanocomposites.

## 1. Introduction

In the past two years, the new crown epidemic has greatly affected people’s health and life and has seriously endangered lives; at the same time, bacteria, fungi, parasites, and viruses are all seriously affecting human public health [1]. As with today’s emphasis on technological innovation, the research and development of new antimicrobial agents that can enhance antibacterial activity is becoming more important in the human living environment [2,3,4], and increased bacterial resistance and cross-infection in public places are also common health threats [5,6,7]. Silver nanoparticles (Ag NPs), which are used as a common antibacterial material, have been extensively studied in the past several decades [8,9], for example, in inhibiting the growth of bacteria [10], antimicrobial activity endurance [11], and the lack of risk of resistance of bacteria [12]. Relevant experiments have demonstrated the antibacterial mechanism of silver, that is, silver ions can cause the denaturation of proteins in the cell membrane of bacteria [13,14,15], and when the Ag^+^ enters into the bacterial cell, it quickly combines with DNA in the cell and prevents the replication of the DNA double-helix structure [16]. It is commonly known that there is an accepted view of nanomaterials in a biological context, that is the smaller the particle size of the nanomaterial, the larger its specific surface area, which, in turn, makes it more biologically active [17,18]. However, the aggregation and potential toxicity of Ag NPs greatly reduce their antibacterial efficiency. It is also reported that high concentrations of silver (1–2.5 μg/mL) can perish human normal cells, for instance, lymphocytes, monocytes, and human mesenchymal stem cells [19,20,21]. It has been reported in some literature that the concentration of silver above 10 μg/mL can be toxic to human cells [22]. Numerous supporting materials have been utilized as basis materials to lower Ag NP concentrations, increase stability, and reduce particle size, including macromolecules [23], photocatalytic materials [24], carbon materials [25], and inorganic metal oxide [26].

Inorganic metal oxides, such as titanium oxide [27], zinc oxide [28], nickel oxide [29], silicon oxide, and aluminum oxide have been increasingly used for antimicrobial applications because of their stability, easy preservation, and low cost [30]. However, the mechanism underlying the antibacterial activity of these metal oxides has not yet been conclusively established. Among the large family of metal oxides, cupric oxide (CuO) [31] is an attractive multifunctional material. It is a p-type semiconductor with a narrow band gap of 1.2 eV and has been reported as a promising material for different applications due to its chemical stability, nontoxic nature, high efficiency, and low production cost [32,33]. In addition, the antibacterial activity of copper ions (Cu^2+^) has been reported by many researchers [34,35] and Cu^2+^ has been reported to possess other valuable biological features such as enhancing the cell activity and proliferation of osteoblastic cells [36], improving the angiogenesis of bioactive scaffolds, wound healing [37], anti-cancer [38,39], and biological imaging probes [40]. Owing to these distinct properties, it is noteworthy to investigate the antimicrobial activities of CuO [41]. In 2018, Azzouz et al. prepared microporous CuO@Ag^0^ core shells with antibacterial activity, especially against *S. aureus* [42]. The next year, Cirandur et al. reported a Nano CuO–Ag, which showed higher antifungal activity against *C. albicans* [43]. Subsequently, Yaya and co-workers also reported CuO/Ag and ZnO/Ag nanocomposites, and both nanocomposites have excellent antibacterial properties against *E. coli* and *S. aureus* [44]. In addition, other CuO and Ag composites have also been reported, which exhibit excellent antibacterial properties [45,46].

Here, we used a simple two-step method to obtain CuO and CuO@Ag nanosheet composites. We investigated the influence of reaction conditions on the morphology and size of the compounds, such as the Cu source, reaction time, pH, and (polyacrylic acid) PAA. The characterization of CuO and CuO@Ag nanosheet composites was achieved using XRD, TEM, and SEM. A range of detection means such as broth dilution, optical density (OD 600), SEM, and TEM were used to assess the antibacterial activities of CuO@Ag nanosheet composites against types of bacteria, for example, Gram-negative bacteria of *E. coli* and *P. aeruginosa*, and Gram-positive bacteria of *S. aureus*.

## 2. Materials and Methods

### 2.1. Reagents

Nantokite (CuSO_4_·5H_2_O, CuNO_3_·3H_2_O, and Cu(CH_3_COO)_2_·H_2_O, purity ≥ 99%), silver nitrate (AgNO_3_, purity ≥ 99%), PAA (1000 (Average), 35 wt.% in H_2_O), sodium hydroxide (purity ≥ 99%), tannic acid (purity ≥ 99%), ammonia hydroxide (NH_3_·H_2_O, 25–28%), and NaCl (purity ≥ 99%) were all bought from Sinopharm (Shanghai, China) and used as-received. Analytical grade was utilized for all mentioned reagents, and they were all used without further purification. Throughout the study, distilled water was used. The nutrients for bacterial growth were provided from AOBOX Biotechnology (Beijing, China). Three types of bacterial strains (*E. coli*, *P. aeruginosa*, and *S. aureus*) were obtained from China General Microbiological Collection Center (CGMCC).

### 2.2. Analytical Methods

The chemical analysis of CuO with different shapes and PAA-regulated silver-carried CuO nanosheet composites were investigated using various techniques: X-ray diffraction (XRD) (Nalytical, Almelo, Holland) using X’pert Philips with Cu Kα radiation (λ = 1.5418 Å); SEM pictures were captured by using a JEOL JSM-5600LV (JEOL Ltd., Tokyo, Japan); transmission electron microscope (TEM) using a JEOL JEM-100CX with the sample carried through a copper mesh; Fourier transform infrared spectrometer (FT-IR) using an AVATAR 360 (Nicolet Instrument Corporation, Madison, USA); UV-vis using a UNICORN 540 (Hefei, Anhui, China); TGA using a Seiko EXSTAR 6000 (Seiko Instruments Inc, Tokyo, Japan) in nitrogen atmosphere.

### 2.3. Preparation of CuO

In this experiment, the reaction vessel was a 250 mL flask, and 1.25 g of CuSO_4_·5H_2_O and 100 mL of distilled water were added into the above reaction vessel to dissolve the blue solution, which was stirred with a magnetic stirrer and heated to 40 °C. Then, 50 mL of 0.3 mol/L NaOH was added dropwise into the reaction, during which the color of the reaction solution changed from blue to black. The solution continued to react for a while. At the end of the process, the suspension liquid was separated by a high-speed centrifuge at 5000 rev/min for 5 min, and the resulting black solid was washed several times with distilled water and then vacuum-dried to obtain a black powder, which was the copper oxide nanosheet. Other shapes of nano-copper oxide were also obtained by a similar procedure as for the copper oxide nanosheet, except that CuSO_4_·5H_2_O was replaced by CuNO_3_·3H_2_O and Cu(CH_3_COO)_2_·H_2_O, respectively.

In addition, some similar experiments were performed, that is, only regulating the reaction time such as 0.5 h, 1 h, 2 h, and 3 h, to observe the morphological changes of copper oxide nanosheets.

### 2.4. Preparation of CuO@Ag

A 90 mL CuO (8.5 mmol/L) solution was added into a 100 mL flask, and the black solution was stirred vigorously with a magnetic stirrer and heated to 40 °C. Then, NaOH (3 mol/L) was slowly added in order to increase the pH value of the reaction solution to 12. Several minutes later, a certain amount of PAA was added and continued to mix for 30 min. After that, a 1 mL solution of silver ammonia (50 mmol/L) and 0.0043 g of tannic acid were added. Finally, the nanocomposites were separated by centrifugation at 8000 rev/min for 5 min, washed several times with distilled water, and vacuum-dried at ambient temperature. The black powder, which is a CuO@Ag nanosheet composite, was obtained.

### 2.5. Antimicrobial Activity Testing

In order to evaluate the antibacterial activity, the operation steps refer to previous literature [47,48]. The target sample was the CuO@Ag nanocomposite, and three types of bacteria such as *E. coli*, *P. aeruginosa*, and *S. aureus* were selected as indicators. For the minimum inhibitory concentration (MIC) test, the concentrations of CuO@Ag nanocomposites were 500, 250, 125, 62.5, 31.3, 15.6, 7.8, 3.9, 2, and 1 μg/mL. Then, the MIC and minimum bactericidal concentration (MBC) of Ag NPs, the CuO@Ag nanosheet composites and CuO nanosheet were obtained.

Another test to investigate the antimicrobial properties of a target sample was the bacterial growth kinetics in broth media. The concentrations of CuO@Ag solutions were 1, 13, and 26 μg/mL, respectively. The detailed steps refer to the literature [49]. Finally, we obtained the growth curve.

In addition, SEM and TEM measurements were performed to assess the morphological changes of bacteria treated with the CuO@Ag nanosheet composite. Amounts of 40 mL of bacterial suspensions and 2 mL of broth medium were combined and cultivated at 37 °C for 6 h. Then, 100 g/mL of CuO@Ag nanosheet composites was introduced, and the bacteria was cultivated for another 6 h under the same conditions. The germs were eventually centrifuged and collected. In order to create bacterial SEM and TEM samples, the bacteria were fixed with a diluted glutaraldehyde solution (2.5%) at −4 °C for 30 min, and then centrifuged at 6000 r/min for 5 min; after being dehydrated using a series of alcohol solutions, the bacteria were collected and examined with SEM and TEM.

## 3. Results and Discussion

### 3.1. XRD Patterns of CuO and CuO@Ag Nanosheet Composites

**Figure 1:**
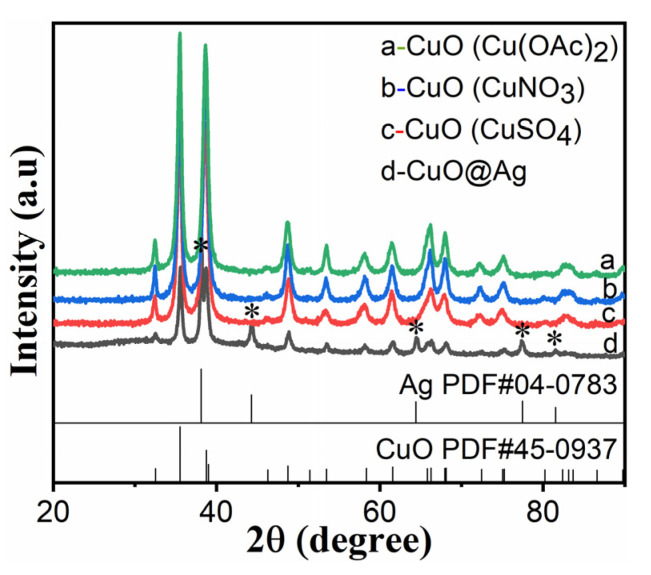
XRD patterns of CuO with different copper sources and CuO@Ag nanosheet composites: (**a**) Cu(CH_3_COO)_2_·H_2_O, (**b**) CuNO_3_·3H_2_O, (**c**) CuSO_4_·5H_2_O, and (**d**) CuO@Ag.

Figure 1 shows the XRD patterns of the CuO by using different copper sources. The three samples present similar multiple diffraction peaks at 32.5°, 35.5°, 38.7°, 48.7°, 53.5°, 58.3°, 61.5°, 66.2°, 67.9°, 72.4°, and 75.2°, which are assigned to diffractions from the (−110), (002), (111), (−202), (020), (202), (−113), (−311), (113), (311), and (−222) lattice planes, respectively. It is confirmed from the International Centre for Diffraction Data (ICDD) card No 450937 and shows that all three samples are copper oxide. However, the curve of d shows more multiple diffraction peaks, in addition to the same peaks of CuO. For example, the peaks (marked “*” in the Figure 1) at 2*θ* of 38.1°, 44.3°, 64.4°, 77.4°, and 81.5° are assigned to diffractions of silver (JCPDS No.04-0783), and these show that this sample has CuO and Ag. In addition, no other impurity peak is observed in the XRD patterns. These results show that all samples are pure phases and they are the CuO and CuO@Ag nanosheet composite, respectively.

### 3.2. Morphologies of CuO Nanosheets

**Figure 2:**
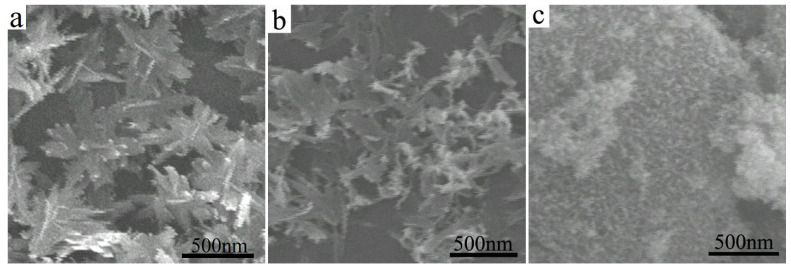
SEM images of CuO nanosheets with different copper sources: (**a**) CuSO_4_·5H_2_O, (**b**) CuNO_3_·3H_2_O, and (**c**) Cu(CH_3_COO)_2_·H_2_O.

Figure 2 shows the SEM images of CuO by using different copper sources: (a) CuSO_4_·5H_2_O, (b) CuNO_3_·3H_2_O, and (c) Cu(CH3COO)_2_·H_2_O. As shown in Figure 2a, the morphology of the sample has a cluster-like pine branch, with an average size of about 400 nanometers. While the morphology of CuO (Figure 2b) uses CuNO_3_·3H_2_O as the copper source is also sheet structure, most of the CuO nanosheet is connected with each other. The sample from Figure 2c has a bulk structure. The samples in Figure 2b,c dot disperse in the water promptly and have some sinkers for a moment compared to the sample from Figure 2a. The results indicate that the copper source plays a crucial role in the morphology and size of the synthesized copper oxide.

**Figure 3:**
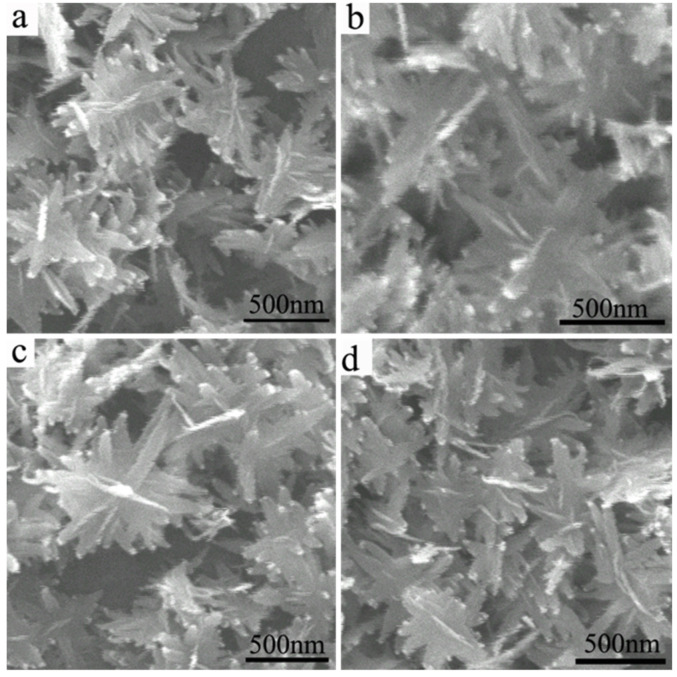
SEM images of CuO nanosheets with different reaction times: (**a**) 0.5 h, (**b**) 1 h, (**c**) 2 h, and (**d**) 3 h.

Figure 3 shows the SEM images of CuO nanosheets with different reaction times: (a) 0.5 h, (b) 1 h, (c) 2 h, and (d) 3 h. The four pictures have too many changes. The morphology of the sample has cluster-like pine branches when the reaction time is 0.5 h (Figure 3a). As the reaction time increases, the cluster-like pine branches of CuO gradually disappear. When the reaction time is 3 h, the morphology of the sample is a separate wide flake (Figure 3d). These results indicate that the reaction time does not have effect on the morphology of the synthesized copper oxide, but the size of the copper oxide becomes larger as the reaction time increases.

### 3.3. Zeta Potential of CuO Nanosheets

**Figure 4:**
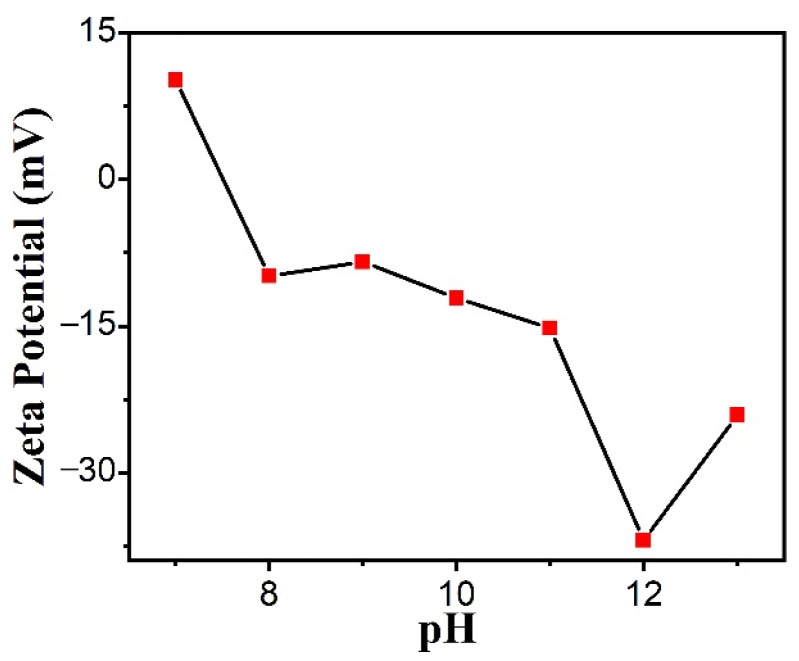
Zeta potential of CuO nanosheet with different values of pH.

Figure 4 shows the zeta potential plotted against pH for the CuO nanosheets. DLS is used to explore the impact of charge on the surface of CuO nanosheets with different pH values [50]. It is clear that the surface of CuO nanosheets is electro-negative in an alkaline environment. As shown in Figure 4, increasing the pH of the nanoparticle suspension leads first to a decrease and then an increase in the absolute value of the zeta potential. The absolute value of the zeta potential on the surface of CuO nanosheets is obtained as maximum when the pH is 12. The maximum absolute value of the zeta potential is 37 mV, which means that the surface of copper oxide has the most negative charge. At the same time, silver ions exist as silver ammonia complex ions rather than as silver salt precipitates when the pH is about 12. The silver ammonia complex ions will accumulate in significant amounts on the surface of CuO nanosheets in accordance with the concept of peer charge repulsion and opposite-charge attraction. The reducing agent will subsequently decrease the silver ammonia complex ions to silver, making it simpler to create CuO@Ag nanosheet composites.

### 3.4. Morphologies of CuO@Ag Nanosheet Composites

**Figure 5:**
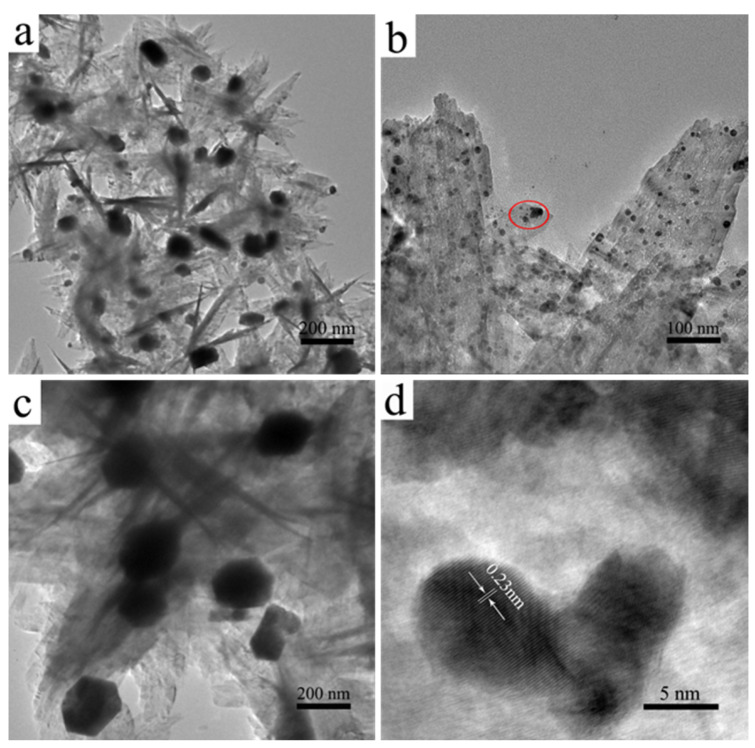
The TEM images of CuO@Ag nanosheet composites: (**a**) aged time: 0 min, without PAA; (**b**) aged time: 1 h, 10% PAA; (**c**) aged time: 1 h, 100% PAA; (**d**) the crystal lattice of the sample in black circle of (**b**).

Figure 5 shows the TEM and HRTEM images of CuO@Ag nanosheet composites with different aging times and surfactants (PAA) while fixing the molar ratio of CuO and Ag^+^: (a) aging time of 0 h, without added PAA modification; (b) aging time of 1 h, 10% PAA; (c) aging time of 1 h, 100% PAA; (d) the crystal lattice of the sample in black circle of (b). From Figure 5a–c, it can be seen that silver NPs are loaded on the surface of the copper oxide nanosheet. From Figure 5d, which is the HRTEM image of the black circle of Figure 5b, there is a clear lattice fringe with a spacing of 0.23 nanometers, which is consistent with the crystal face (111) of face-centered cubic (fcc) silver (JCPDS No.04-0783); silver nanoparticles are about 3 nm; combined with the results of XRD, it further confirms that the silver nanoparticles are loaded on the surface of copper oxide nanosheets. From Figure 5b, it can be seen clearly that there are lots of small silver nanoparticles loading equally on the surface of the CuO nanosheet. When the reaction liquid is separated immediately, there are several large silver nanoparticles unevenly loading on the surface of copper oxide (as shown in Figure 5a); the same phenomenon also appears in Figure 5c. It may be due to the connection between superfluous PAA and making silver nanoparticles easily reunite. This result is consistent with the results of Ag/PAA nanoparticles. These results show that the aging time of 1 h and 10% PAA are the best conditions to obtain a suitable CuO@Ag nanosheet composite when other conditions are constant.

### 3.5. Antimicrobial Activities of CuO@Ag Nanosheet Composites

According to the broth dilution method, Table 1 displays the MIC and MBC values of Ag, CuO@Ag nanosheet composites, and CuO nanosheets against three types of bacteria, for instance, *E. coli*, *S. aureus*, and *P. aeruginosa*. The average particle diameters of CuO@Ag nanosheet composites and Ag NPs on the surface of the CuO nanosheet are 400 nm and about 20 nm, respectively, which are prepared by the two-step method in this work. Yet, Ag particles with severe aggregation are about 400 nm and used as the control sample. Table 1 shows that the MIC and MBC values of Ag and CuO nanosheets against the three bacteria are more than 125 μg/mL. While obviously superior to those of Ag and CuO nanosheets, the MIC and MBC values of CuO@Ag nanosheet composites against three types of bacteria (*E. coli*, *S. aureus*, and *P. aeruginosa*) are lower than 13 and 26 μg/mL, respectively. These findings demonstrate that the CuO@Ag nanosheet composites have superior antibacterial qualities. According to Table 1, the results also suggest that the CuO@Ag nanosheet composites display comparable antibacterial properties against Gram-negative bacteria such as *P. aeruginosa* and *E. Coli*, but this sample differs from the reference in that it shows the higher antibacterial activity against Gram-positive bacteria such as *S. aureus* [51]. This could be explained by the outcome of the combined action of Ag NPs and CuO nanosheets.

**Figure 6:**
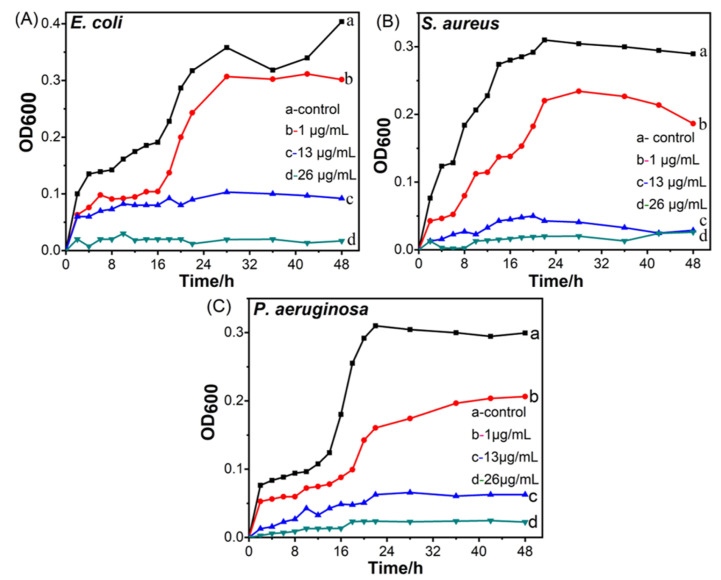
Growth curves of *E. coli* (**A**), *S. aureus* (**B**), and *P. aeruginosa* (**C**) treated with different concentrations of CuO@Ag nanosheets (**b**–**d**) and normal bacterial as control test (**a**).

In addition, we investigate the antibacterial properties of CuO@Ag nanosheet composites by testing the bacterial growth curves in liquid broth media. Using a UV-vis spectrophotometer, the time-dependent variations in bacterial growth are identified using the OD600 technique. The growth curves of common Gram-negative and Gram-positive bacteria (*E. coli* and *P. aeruginosa*) are shown in Figure 6 for 48 h with varying concentrations of CuO@Ag nanosheet composites. The control test uses untreated normal bacteria. Figure 6 shows that CuO@Ag nanosheet composites have a considerable inhibitory effect on the reproduction of the tested strains at all tested doses. When the concentration exceeds 26 g/mL, the sample can totally block the growth of *P. aeruginosa* and *E. coli* for the full 48 h (Figure 6A,C). The growth of *E. coli* and *P. aeruginosa* is delayed when the concentration is below the MIC (13 g/mL), as it is insufficient to stop their growth within 48 h. The 13 and 26 g/mL solutions may totally stop the growth of *S. aureus* bacteria. Similar to the control test of *S. aureus*, the growth curves of *S. aureus* with the concentration of 2 g/mL of CuO@Ag nanosheet composites similarly display a lag phase (Figure 6B). These results are consistent with the MIC and MBC numerical values.

**Figure 7:**
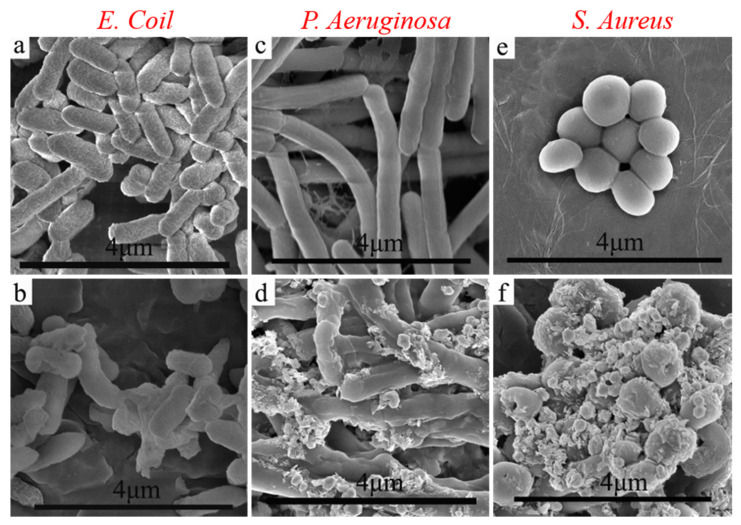
SEM images of the normal and treated bacteria with CuO@Ag solution (100 μg/mL) for *E. coli* (**a**,**b**), *P. aeruginosa* (**c**,**d**), and *S. aureus* (**e**,**f**).

We use SEM to compare the appearance of the normal and treated bacteria in order to further understand how the CuO@Ag nanosheet composites solution affects bacteria. Figure 7 presents SEM images of the three types of bacteria before and after being treated with the CuO@Ag nanosheet composites solution (100 μg/mL). In contrast to the normally occurring *E. coli*, which exhibits a uniformly short rod with a smooth surface (Figure 7a), the treated *E. coli* (Figure 7b) shows significant differences, with shorter lengths and an extremely rough surface. Similarly, both *P. aeruginosa* and *S. aureus* show similar phenomena. That is, the normal *P. aeruginosa* (Figure 7c) presents a uniformly long rod with a smooth surface, while the treated *P. aeruginosa* (Figure 7d) has a highly rough and uneven surface. The normal *S. aureus* cells (Figure 7e) have a smooth surface and spherical shape with an average diameter of 1 μm; however, in treated cells, cell debris can be visible along with membrane distortion and rough surface development (Figure 7f). These results suggest that nanocomposites interact with and destroy bacteria, rendering them inactive.

**Figure 8:**
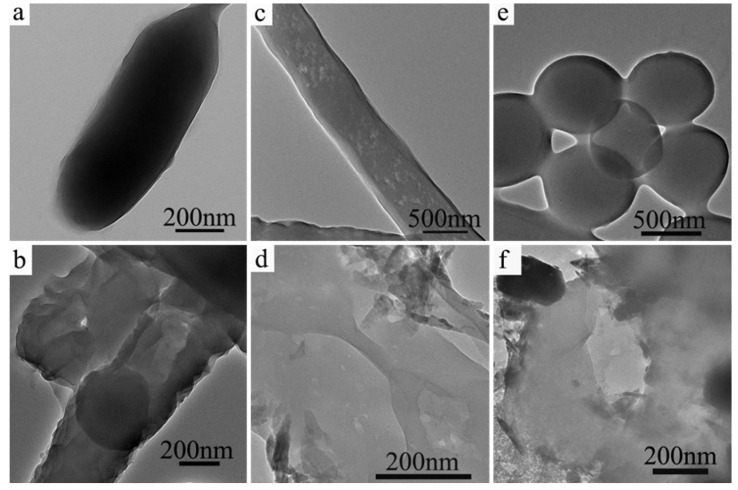
TEM images of normal *E. coli* (**a**), *P. aeruginosa* (**c**), *S. aureus* (**e**), and *E. coli* (**b**), *P. aeruginosa* (**d**) and *S. aureus* (**f**) treated with CuO@Ag nanosheets.

To further demonstrate the mechanism of CuO@Ag nanosheet composites and bacteria, we also use TEM to observe the morphological changes of the three bacteria, *E. coli*, *P. aeruginosa* and *S. aureus*. Figure 8 shows TEM images of the normal bacteria *E. coli* (a), *P. aeruginosa* (c), *S. aureus* (e) without CuO@Ag nanosheet composites and the treated bacteria *E. coli* (b), *P. aeruginosa* (d), *S. aureus* (f) with CuO@Ag nanosheet composites (100 μg/mL). It is obvious to see from Figure 8 that the normal bacteria are rod-like and spherical for *E. coli*, *P. aeruginosa*, and *S. aureus*, respectively. After being treated with CuO@Ag nanosheet composites, *E. coli*, *P. aeruginosa*, and *S. aureus* are all broken, and the antibacterial agents CuO@Ag nanosheet composites adhere to or around the bacteria. Based on the above results, the possible antibacterial model of CuO@Ag nanosheet composites against bacteria can be proposed. That is, CuO@Ag nanosheet composites can attach to the surface of the cell membrane, thus reducing the stability of the cell membrane. The copper and silver could cause more severe membrane disruption, and then enter the bacteria, resulting in massive cytoplasmic efflux. In addition, CuO@Ag nanosheet composites may release highly concentrated Ag^+^ and Cu^2+^, and these high concentrations of metal ions exacerbate the death of bacteria [45,52].

## 4. Conclusions

In this article, a variety of copper oxide (CuO) with different shapes and PAA regulated silver-carried CuO (CuO@Ag) nanosheet composites have been successfully synthesized by a facile and green aqueous solution approach. Both CuO and CuO@Ag have a laminar structure and exhibit good crystallization, and the copper source and reaction duration have a sizable impact on the morphology and size distribution of CuO. In the process of synthesizing CuO@Ag nanosheet composites, the appropriate amount of polyacrylic acid (PAA) can inhibit the agglomeration of Ag NPs and regulate the size of Ag. In addition, compared with silver NPs and copper oxide nanosheets, the CuO@Ag nanosheet composites exhibit excellent synergistic and antibacterial action, particularly against *S. aureus*. This indicates that the CuO@Ag nanosheet composites have a synergistic effect on the antibacterial efficiency of three bacteria. The antimicrobial mechanism of the CuO@Ag nanosheet composites can be attributed to the destruction of the bacterial cell membrane and the consequent leakage of the cytoplasm by the release of Ag^+^ and Cu^2+^. The breakdown of the bacterial cell membrane and subsequent leakage of cytoplasm caused by Ag^+^ and Cu^2+^ released from antimicrobial agents may be the cause of the CuO@Ag nanosheet composites’ antibacterial action. This study can provide the basis and ideas for the application research of other silver nanocomposites.

## Figures and Tables

**Table 1:** MIC and MBC numerical values of Ag, CuO@Ag nanocomposites, and CuO nanosheets.

	MIC (μg/mL)			MBC (μg/mL)		
	*E. coli*	*P. aeruginosa*	*S. aureus*	*E. coli*	*P. aeruginosa*	*S. aureus*
Ag NPs	125	250	125	250	500	250
CuO@Ag	13	6.5	3.2	26	26	13
CuO nanosheet	500	500	125	1000	1000	500

## Data Availability

All data are presented in the manuscript.
